# Experimental Demonstration of a Highly Efficient Fan-out Polarization Grating

**DOI:** 10.1038/srep39626

**Published:** 2016-12-23

**Authors:** Chenhao Wan, Jian Chen, Xiahui Tang, Qiwen Zhan

**Affiliations:** 1 School of Optical and Electronic Information, Huazhong University of Science and Technology, Wuhan, Hubei 430074, China; 2 Department of Electro-Optics and Photonics, University of Dayton, 300 College Park, Dayton, Ohio 45469, USA; 3 School of Electronic Engineering, University of Electronic Science and Technology of China, Chengdu, Sichuan 611731, China

## Abstract

Highly efficient fan-out elements are crucial in coherent beam combining architectures especially in coupled laser resonators where the beam passes through the fan-out element twice per round trip. Although the theoretical efficiency is usually less than 86%, the Dammann gratings are ubiquitously utilized in a variety of types of coherent beam combining systems due to the facile design and fabrication. In the current paper, we experimentally demonstrate a highly efficient fan-out polarization grating. It is the first time to our knowledge that all the three space-variant parameters of a polarization grating are simultaneously optimized to achieve the function of multi-beam splitting. Besides the high fan-out efficiency, the ability to control the polarization states of individual split beams is another advantage of this polarization grating. The novel polarization grating is promising to find applications in laser beam combining systems.

Increasing the radiance of laser systems is a long-term goal to research scientists in pursuit of broadening the range of laser applications. Conventional single gain element lasers are limited in the amount of power they can produce by optical damage, nonlinear effects and thermally induced distortions. Beam combining techniques provide an alternative to higher radiance by combining the power from a number of lower power gain elements while maintaining the spectral and polarization features of the individual lasers.

A highly efficient fan-out element is always desirable in beam combining systems. Dammann gratings are the most common and widely used fan-out element in a variety of laser combining systems such as quantum-cascade lasers^1^, diode lasers^2^, fiber lasers^3^ and solid state lasers^4^,^5^. Dammann gratings are facile to design and fabricate due to the binary phase features; however, the theoretical efficiency is lower than 86%^6^.

Unlike conventional phase gratings which modulate only the phase with surface relief profiles or refractive index variation, the polarization grating controls both the phase and the amplitude of the two orthogonally polarized components^7^,^8^. The amplitude modulation is achieved by exchanging energy between the two orthogonal polarizations and the total energy is preserved. Since the optimization of the two orthogonal polarizations is exploited, fan-out polarization gratings possess higher theoretical efficiencies which can approach 100%^9^,^10^,^11^,^12^. The design of fan-out polarization gratings either optimizes the space-variant azimuth angle^13^,^14^ or the space-variant phase retardation^10^,^15^,^16^ to uniformly distribute energy into several diffraction orders.

Recently we proposed a novel scheme that exploits all three parameters, namely the space-variant azimuth angle, the space-variant relative phase retardation and the space-variant phase bias, to design a polarization diffraction grating in the reverse direction of beam propagation^17^. The advantage of the scheme is that not only can we achieve highly efficient and uniform beam splitting but also we can control the polarization states of the individual fan-out beams.

In the optimization process, the polarization states of the individual split beams in the Fourier plane are exploited as the optimization parameters and their combined intensity in the pupil plane is optimized to match the intensity of the incident beam. A polarization diffraction grating is designed via this method to split an incident linearly polarized beam to an array of linearly polarized beams with equal intensity and various linear polarization angles.

In the current paper, we experimentally demonstrate this novel scheme with the use of a vector beam generator based on high-resolution reflective phase-only liquid crystal spatial light modulators (SLM). We perform laser beam splitting for the case of a duplicator, a triplicator and a 1-by-4 splitter. The purpose of this paper is to present an experimental demonstration of the validity of the fan-out polarization grating design. The actual gratings should be fabricated with metasurfaces^18^,^19^ or form-birefringence structures^20^ which would be compact, robust and highly efficient. The high efficiency and the ability of controlling the individual polarization states of split beams make this fan-out polarization grating useful to laser beam combining systems.

## Results

### Theoretical design of the highly efficient fan-out polarization grating

The fan-out polarization grating applies space-variant modulation of the polarization state to an incident beam and splits the beam into an array of beams with equal intensity in the Fourier plane. The polarization grating is characterized by three space-variant parameters: the azimuth angle φ, the relative phase retardation Γ and the phase bias α. The azimuth angle φ is the angle between the x axis and the local slow axis; the relative phase retardation Γ is the optical phase difference between the local slow axis and fast axis; the phase bias α is the common phase. The transmission Jones matrix of a polarization grating that is space-variant in the x-direction is given by^21^,^22^:


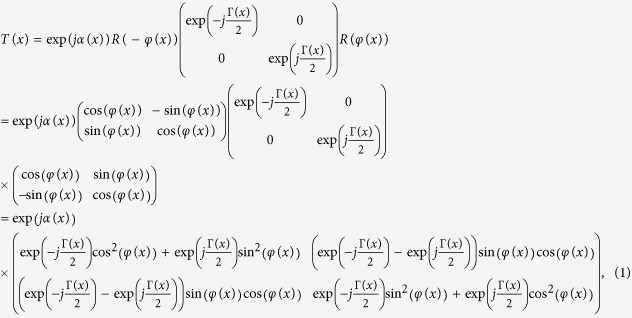


where R is the rotation matrix. The beam incident on the polarization grating is x-polarized and its Jones vector is 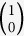. The transmitted beam is represented by the Jones vector:


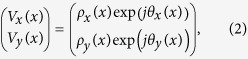


where ρ_x_(x) and ρ_y_(x) are the local amplitude of the x and y polarization components whereas θ_x_(x) and θ_y_(x) are the local phase of the x and y polarization components. Combining equation (1) and (2) yields:


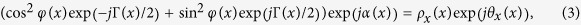



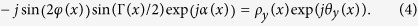


**Figure 1:**
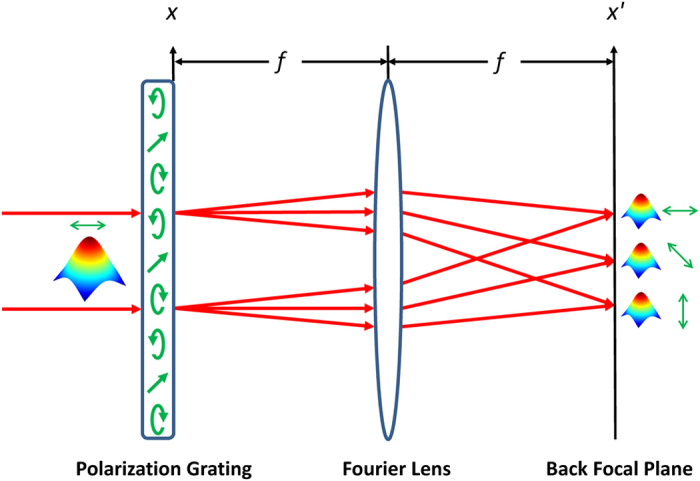
Schematic of beam splitting by the fan-out polarization grating.

The polarization grating is placed in the front focal plane of a Fourier lens and the Fraunhofer diffraction pattern is obtained in the back focal plane of the lens as illustrated in Fig. 1. The x′-polarization component V_x′_(x′) and y′-polarization components V_y′_ (x′) of the optical field in the back focal plane are Fourier transform related to the x-polarization component V_x_(x) and y-polarization component V_y_(x) of the grating transmitted field in the front focal plane respectively. The optical field in the back focal plane consists of an array of Gaussian beams with identical intensity distribution and various linear polarization angles. The design and optimization of the polarization grating is performed in the reverse direction of beam propagation. By tuning the linear polarization angles of the individual Gaussian beams in the back focal plane, their combined intensity distribution in the front focal plane is optimized to match the intensity of a single Gaussian beam. Comparing the change of the local polarization states between the incident and transmitted field, the three space-variant parameters of the polarization grating can be derived accordingly.

After the optimization process, the numerical expressions of V_x_(x) and V_y_(x), or ρ_x_(x), ρ_y_(x), θ_x_(x) and θ_y_(x) are obtained. Therefore, the three parameters of the fan-out polarization grating (the phase bias α(x), the relative phase retardation Γ(x) and the azimuth angle φ(x)) can be derived from equations (3) and (4) afterwards:


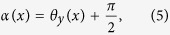



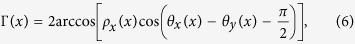



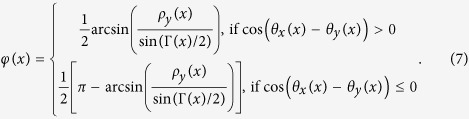


### The experimental setup of the fan-out polarization grating

**Figure 2:**
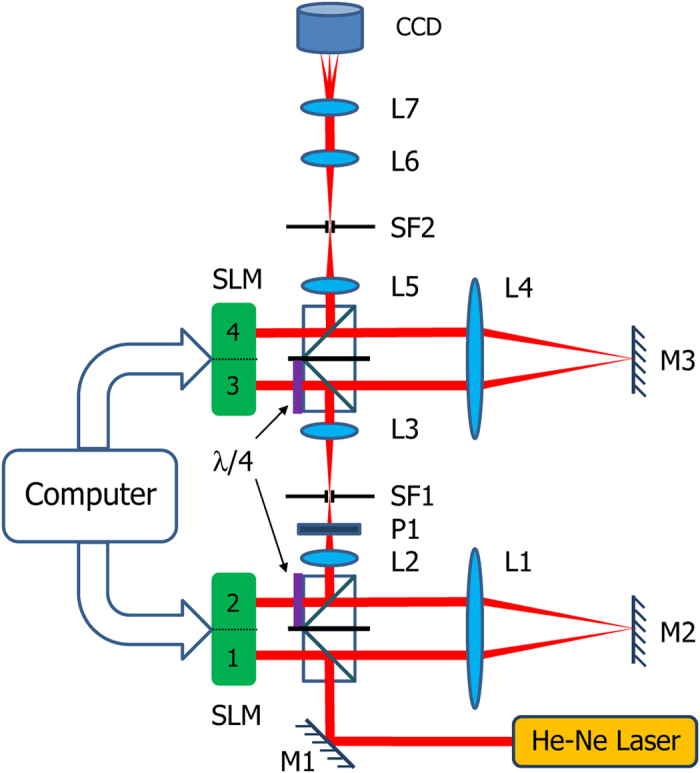
The diagram of the fan-out experiment setup.

Given the three space-variant parameters (the phase bias α(x), the relative phase retardation Γ(x) and the azimuth angle φ(x)) expressed in equation (5)–(7), the fan-out polarization grating can be fabricated and physically realized via metasurfaces^18^,^19^ or form-birefringence structures^20^. In the current paper, we perform a demonstration with the use of a vector beam generator^23^ to produce the transmitted field 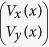 of the polarization grating, and measure the intensity distribution and polarization states in the back focal plane of a Fourier lens L7 as shown in Fig. 2.

The diagram of the fan-out experiment is presented in Fig. 2. The He-Ne laser produces an x-polarized beam. The beam is reflected by the mirror M1 and a cube beam splitter, and propagates to the left half of a spatial light modulator (SLM). The left half of the SLM is denoted as section 1 in Fig. 2. The right half of the SLM is labeled as section 2. The left and right halves of the other SLM are referred to as section 3 and section 4 in Fig. 2. The SLM is phase-only and responsive only to the x-polarization component. Therefore, the x-polarized beam obtains a controllable phase from section 1 of the SLM. The optical field is then relayed to section 2 of the SLM with a folded 4 f system that consists of lens L1 and mirror M2. The beam transmits through a quarter wave plate, hits section 2 of the SLM and passes through the quarter wave plate again. The quarter wave plate is aligned 45 degrees to the x-axis. With the use of the folded configuration of a quarter wave plate and section 2 of the SLM, the linear polarization angle of the beam can be controlled by the phase applied on section 2^23^. The beam is then reflected by another cube beam splitter and passes through a 4 f system with a polarizer P1 and a spatial filter SF1 along the path. The polarizer is aligned to the x-direction. Since the phase on section 2 controls the linear polarization angles of the beam before the polarizer P1, equivalently, the phase on section 2 controls the amplitude of the beam after the polarizer P1. The spatial filter SF1 is utilized to filter out higher order diffractions. Note that a piece of black paper is sandwiched between the two cube beam splitters to prevent from beam propagation in unwanted directions. The beam then propagates to section 3 through the 4 f system formed by lens L2 and L3. The similar folded configuration of a quarter wave plate and section 3 of the SLM is exploited to adjust the linear polarization angles of the beam. In other words, energy is redistributed to x and y components by the phase on section 3. The beam is relayed again to section 4 by a folded 4 f system that consists of lens L4 and mirror M3. Section 4 of the SLM controls the relative phase between the x and y components by adjusting the phase of the x components.

All the four sections work in a cascaded way along the direction of beam propagation and are capable of creating the complex transmitted field of the fan-out polarization grating on a pixel by pixel level. Section 1 applies the common phase θ_x_(x) to the incident x-polarized beam. Section 2 is utilized to confine the size of the beam to a diameter of 3 mm. The phases on section 2 are modulated so that the transmittance is unity inside a circle of 3 mm in diameter and zero outside the circle. Section 3 is exploited to rotate the linear polarization angles of the beam by tan^−1^(ρ_y_(x)/ρ_x_(x)). Section 4 introduces the relative phase retardation between the x and y polarization components −(θ_y_(x) − θ_x_(x)). Since the phase modulation by the SLM is applicable only to the x-polarized component, a minus sign is added to (θ_y_(x) − θ_x_(x)). After the sequential modulations by each section of the SLM panels, the transmitted field of the fan-out polarization grating is generated in the plane of section 4 and relayed by a 4 f system formed by lens L5 and L6 to the back focal plane of lens L6. The Fourier lens L7 is placed between L6 and the CCD camera. The camera is positioned in the back focal plane of L7 and measures the far-field intensity distribution and polarization states.

### Experimental results of the beam splitting by a duplicator, triplicator and 1-by-4 splitter

**Figure 3:**
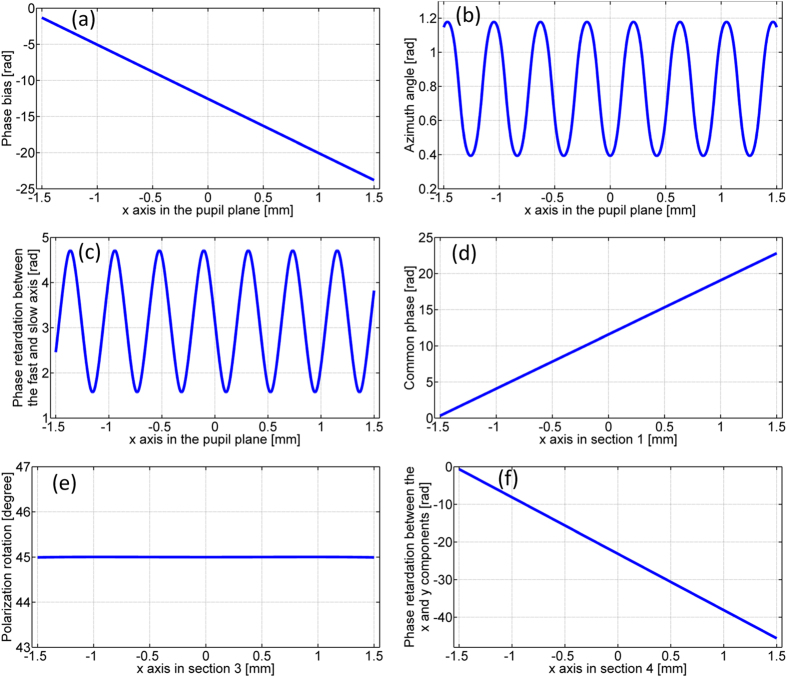
The three space-variant parameters of a duplicator and the loaded phase patterns on section 1, 3 and 4 for generating the transmitted field of the duplicator (**a**) The phase bias α(x). (**b**) The azimuth angle φ(x). (**c**) The phase retardation Γ(x) between the fast and slow axis. (**d**) The common phase θ_x_(x) applied on section 1. (**e**) The polarization rotation tan^−1^(ρ_y_(x)/ρ_x_(x)) accomplished by section 3. (**f**) The phase retardation −(θ_y_(x) − θ_x_(x)) between the x and y components (section 4).

The simplest case of a fan-out polarization grating is a duplicator that splits an x-polarized beam into two linearly polarized beams of orthogonal polarizations. The linear polarization angles of the two split beams are found to be 0 and 90 degrees from the optimization process. The grating parameters (the phase bias α(x), the phase retardation Γ(x), and the azimuth angle φ(x)) are given by equation (5) to (7), and plotted in Fig. 3(a) to (c) respectively. Note that all the three grating parameters and the loaded phase patterns on each section of the SLMs are two-dimensional and space-variant only in the x-direction. Therefore only the one-dimensional data along the x-direction are plotted throughout the rest of the paper.

**Figure 4:**
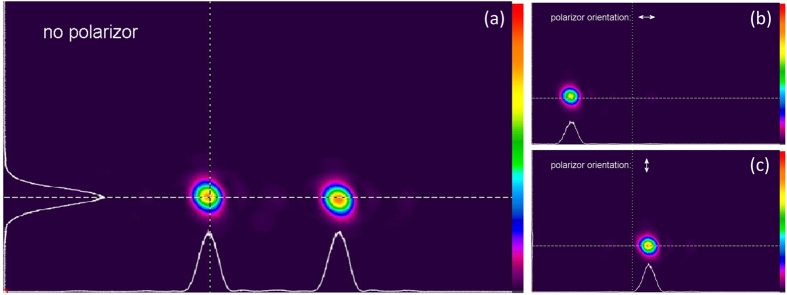
Far-field intensity distribution in the back focal plane of lens L7. (**a**) With no polarizer in front of the camera; (**b**) when the polarizer is oriented horizontally in front of the camera; (**c**) when the polarizer is oriented vertically in front of the camera.

The phase patterns loaded on section 1, 3 and 4 of the SLM panels for generating the transmitted field of the duplicator are the common phase θ_x_(x), the polarization rotation tan^−1^(ρ_y_(x)/ρ_x_(x)) and the phase retardation between the x and y polarization components − (θ_y_(x) − θ_x_(x)) which are plotted in Fig. 3(d) to (f) respectively. The Fourier lens L7 is placed between the lens L6 and the camera. The camera is positioned in the Fourier plane and measures the far-field intensity distribution and polarization states. Figure 4(a) shows the intensity distribution in the back focal plane of lens L7 with no polarizer in front of the camera. By placing and rotating a polarizer in front of the camera, we demonstrate that both spots are linearly polarized and the linear polarization angles are 0 and 90 degrees, as shown in Fig. 4(b,c). Figure 4(a,b,c) use the same color scale bars. The spots in Fig. 4(b,c) appear less bright than the spots in Fig. 4(a) due to the Fresnel reflection from the polarizer surfaces.

**Figure 5:**
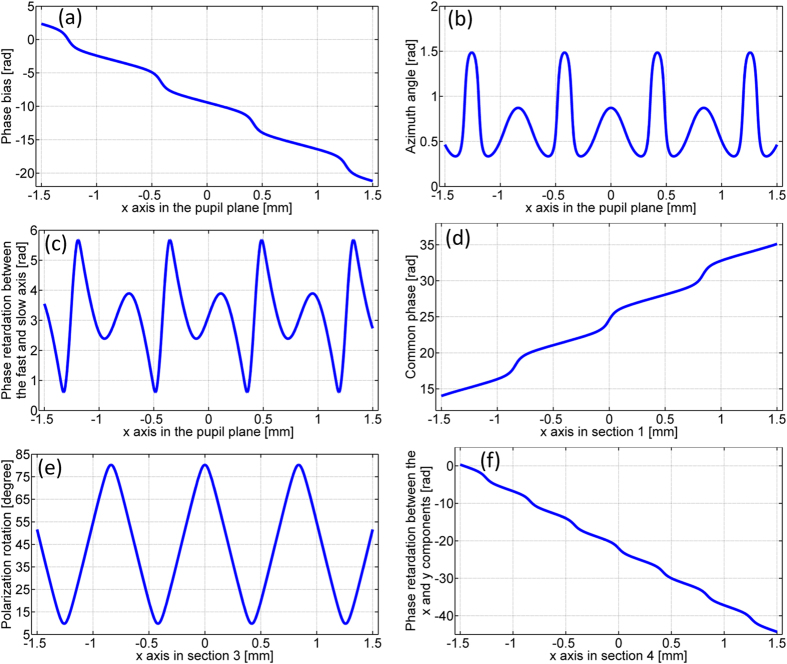
The three space-variant parameters of a triplicator and the loaded phase patterns on section 1, 3 and 4 for generating the transmitted field of the triplicator. (**a**) The phase bias α(x). (**b**) The azimuth angle φ(x). (**c**) The phase retardation Γ(x) between the fast and slow axis. (**d**) The common phase θ_x_(x) applied on section 1. (**e**) The polarization rotation tan^−1^(ρ_y_(x)/ρ_x_(x)) accomplished by section 3. (**f**) The phase retardation −(θ_y_(x) − θ_x_(x)) between the x and y components (section 4).

The second example is a triplicator that splits an x-polarized beam into three spots with the linear polarization angles of 0, 45 and 90 degrees. The three space-variant grating parameters are plotted in Fig. 5(a) to (c) and the patterns displayed on section 1, 3 and 4 of the SLM panels for generating the transmitted field of the triplicator are shown in Fig. 5(d) to (f).

**Figure 6:**
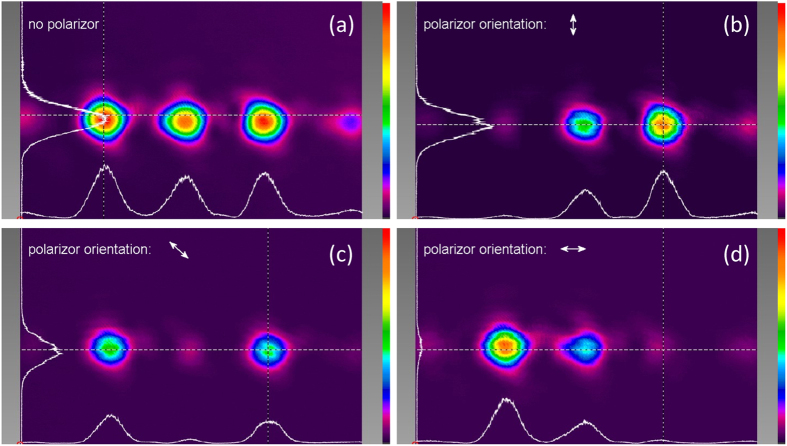
Far-field intensity distribution in the back focal plane of lens L7. (**a**) With no polarizer in front of the camera; (**b**) when the polarizer is aligned vertically in front of the camera; (**c**) when the polarizer is oriented at 135 degrees in front of the camera; (**d**) when the polarizer is aligned horizontally in front of the camera.

Figure 6(a) shows three spots with equal intensity captured in the back focal plane of lens L7 by the camera. If a polarizer is aligned vertically (Fig. 6b) or horizontally (Fig. 6d) and placed in front of the camera, the intensity of the middle spot is decreased by half, and either the left or the right spot disappears and the other one remains at its maximum intensity. Figure 6(c) shows the intensity distribution when the polarizer is oriented at 135 degrees. The intensity of the left and right spots is reduced by half and the middle spot disappears. Figure 6(b,c,d) use the same color scale bars and these three figures demonstrate the polarization states of the spots. Figure 6(a) demonstrates the intensity distributions of the spots. The color scale bars used in Fig. 6(b,c,d) has a lower maximum value than the color scale bar used in Fig. 6(a) so that the spots at their half maximum intensity would appear more observable in Fig. 6(b,c,d). The experimental results are consistent with the theoretical polarization states and intensity distributions.

**Figure 7:**
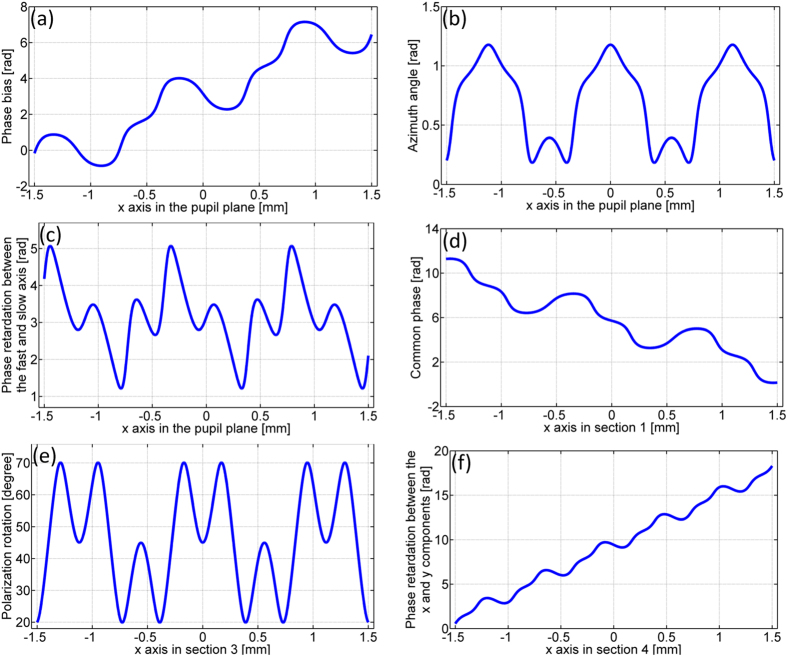
The three space-variant parameters of a 1-by-4 splitter and the loaded phase patterns on section 1, 3 and 4 for generating the transmitted field of the 1-by-4 splitter. (**a**) The phase bias α(x). (**b**) The azimuth angle φ(x). (**c**) The phase retardation Γ(x) between the fast and slow axis. (**d**) The common phase θ_x_(x) applied on section 1. (**e**) The polarization rotation tan^−1^(ρ_y_(x)/ρ_x_(x)) accomplished by section 3. (**f**) The phase retardation −(θ_y_(x) − θ_x_(x)) between the x and y components (section 4).

The third example is a 1-by-4 beam splitter that splits an x-polarized beam into four spots with the linear polarization angles of 45, 45, 135 and 135 degrees. The three grating parameters are plotted in Fig. 7(a) to (c) and the patterns displayed on section 1, 3 and 4 of the SLM panels for generating the transmitted field of the 1-by-4 splitter are shown in Fig. 7(d) to (f).

**Figure 8:**
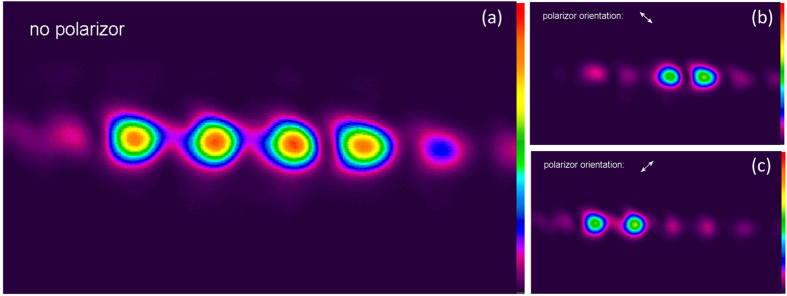
Far-field intensity distribution in the lens focal plane. (**a**) With no polarizer in front of the camera; (**b**) when the polarizer is oriented at 135 degrees in front of the camera; (**c**) when the polarizer is oriented at 45 degrees in front of the camera.

Figure 8(a) displays four spots with equal intensity captured in the back focal plane of lens L7 by the camera. If a polarizer is oriented at 135 degrees, the left two spots disappear (Fig. 8b); if a polarizer is oriented at 45 degrees, the right two spots vanish (Fig. 8c). Figure 8(a,b,c) use the same color scale bars. The spots in Fig. 8(b,c) are less bright than the spots in Fig. 8(a) due to the Fresnel reflection from the polarizer surfaces.

One design parameter of the fan-out polarization grating that differ among all three examples is the spacing between adjacent spots. For the case of a duplicator, a triplicator, and a 1-by-4 splitter, we choose 8ω_0_, 4ω_0_, and 3ω_0_ as the spot spacing, where ω_0_ is the half width of the individual split beams. The further apart the adjacent spots, the higher spatial frequency components in the complex optical field. 2ω_0_ is not a great option for the spot spacing because the spots become noticeably overlapped.

**Figure 9:**
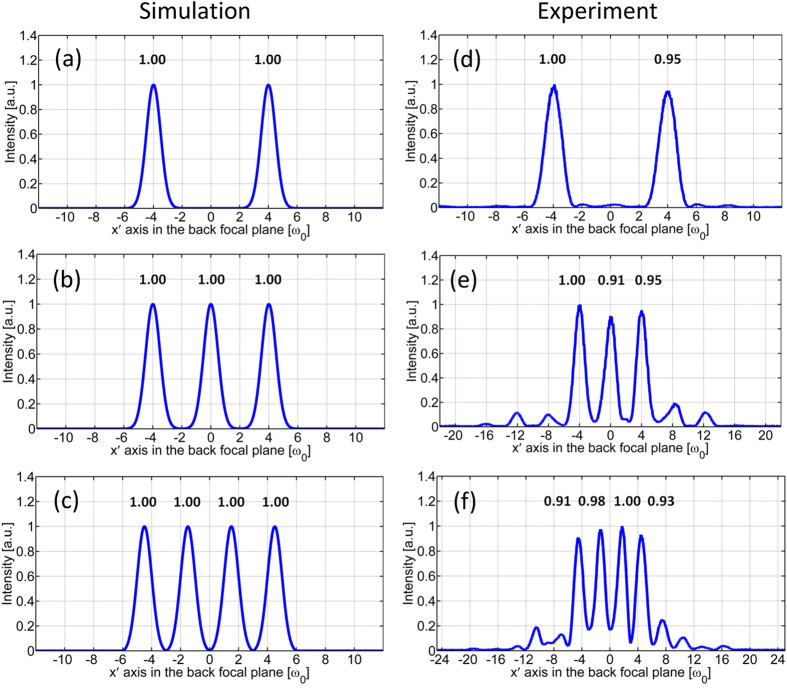
Far-field intensity distribution along the x′-axis in the lens focal plane with no polarizer in front of the camera. (**a**) Simulation of a duplicator; (**b**) simulation of a triplicator; (**c**) simulation of a 1-by-4 splitter; (**d**) experimental result of a duplicator; (**e**) experimental result of a triplicator; (**f**) experimental result of a 1-by-4 splitter.

Fan-out polarization gratings simultaneously modulate both the phase and the amplitude of two orthogonal electric-vector components which results in the suppression of undesired orders^9^. Therefore highly efficient and uniform beam splitting can be achieved with fan-out polarization gratings. Given the polarization grating parameters plotted in Figs 3(a–c), 5(a–c) and 7(a–c), it is straightforward to calculate the transmitted field of the polarization grating through equation 1 and the far-field intensity distribution through Fraunhofer diffraction. Figure 9(a,b,c) show the simulated intensity distribution in the back focal plane with no polarizer in front of the camera for the case of a duplicator, a triplicator and a 1-by-4 splitter. Figure 9(d,e,f) are one-dimensional experimental results line-scanned in the center along the x’-axis from Figs 4(a), 6(a) and 8(a) respectively. The fan-out efficiency is defined as the ratio of the power of desired orders to the power of all orders. The theoretical efficiency of a duplicator, a triplicator and a 1-by-4 splitter are 100% as demonstrated in Fig. 9(a) to (c). The experimental efficiency of a duplicator, a triplicator and a 1-by-4 splitter are 97.5%, 84.3% and 82.6% as shown in Fig. 9(d) to (f). The fan-out uniformity is defined as the ratio between the minimal and the maximal intensity within the array^20^. The theoretical uniformity of a duplicator, a triplicator and a 1-by-4 splitter are unity as demonstrated in Fig. 9(a) to (c). The experimental uniformity of a duplicator, a triplicator and a 1-by-4 splitter are 0.95, 0.91 and 0.91 as shown in Fig. 9(d) to (f).

We would like to emphasize that the purpose of the experiment is to demonstrate the validity of the polarization grating design. The physical realization of the fan-out polarization gratings is to be achieved with the use of metasurfaces^18^,^19^ or form-birefringence structures^20^. These devices accomplish the spatial polarization modulation not in a cascaded way but in one step. They do not suffer from the problems such as misalignment of a complex optical system, phase inaccuracy of the SLM panels, and distortions caused by 4 f relay systems. With the advance in nanotechnology, these single-piece fan-out polarization gratings are expected to approach the theoretical values of both the efficiency and uniformity.

## Discussion

**Figure 10:**
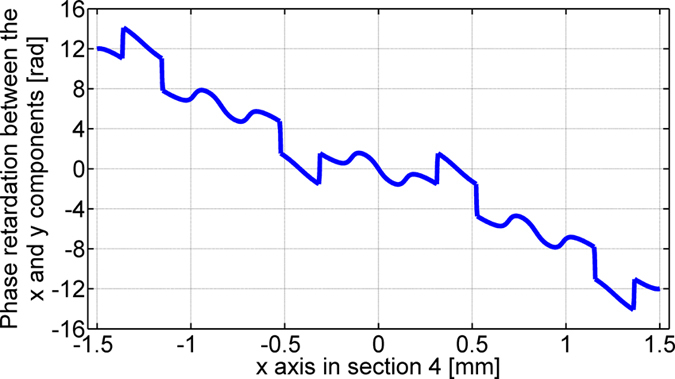
The phase retardation between the x and y components (section 4).

As the number of split spots increases, the uniformity of the spots begins to deteriorate and the polarization states also deviate from the theory. We notice that abrupt phase jumps emerge in the patterns loaded on the three sections of the SLM panels. Figure 10 shows the data −(θ_y_ − θ_x_) loaded on section 4 for the case of a 1-by-5 splitter. Abrupt phase jumps indicate high spatial frequency components exist in the data which may not be properly relayed by the 4 f systems.

**Figure 11:**
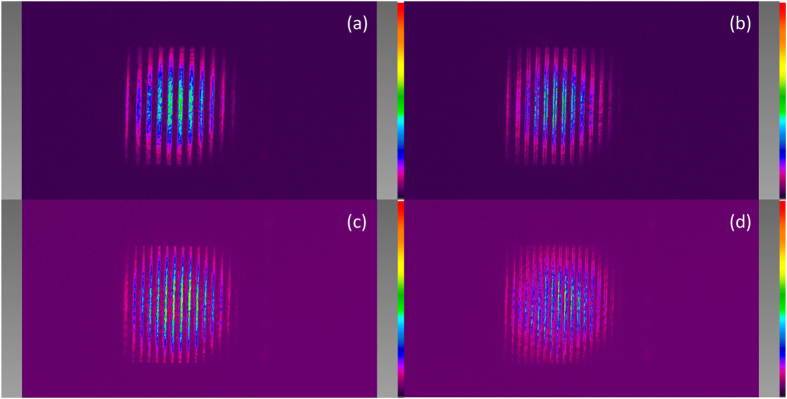
Intensity distribution in the back focal plane of L6. (**a**) When the period of the amplitude grating is 256 μm; (**b**) when the period of the amplitude grating is 224 μm; (**c**) when the period of the amplitude grating is 192 μm; and (**d**) when the period of the amplitude grating is 160 μm.

To investigate the maximum spatial frequency that can propagate accurately through the 4 f relay systems, we display a set of amplitude gratings with different periods on section 2 of the SLM panels and measure the intensity distributions in the back focal plane of lens L6. Figure 11(a) to (d) show the intensity distributions in the back focal plane of L6 when the period of the amplitude grating is 32 pixels, 28 pixels, 24 pixels and 20 pixels, i.e., 256 μm, 224 μm, 192 μm and 160 μm respectively. The size of each pixel of the SLM is 8 μm. The grating pattern captured by the camera becomes blurrier as the period of the grating decreases. We estimate the cutoff spatial frequency of the 4 f system is around 1/(22 pixel width), i.e., 5.6 mm^−1^. As the contrast of the fringes deteriorates, the maximum intensity of the fringes keeps decreasing from Fig. 11(a) to (d). The color scale bars are adjusted among these figures so that the fringes are sufficiently bright to be observed.

**Figure 12:**
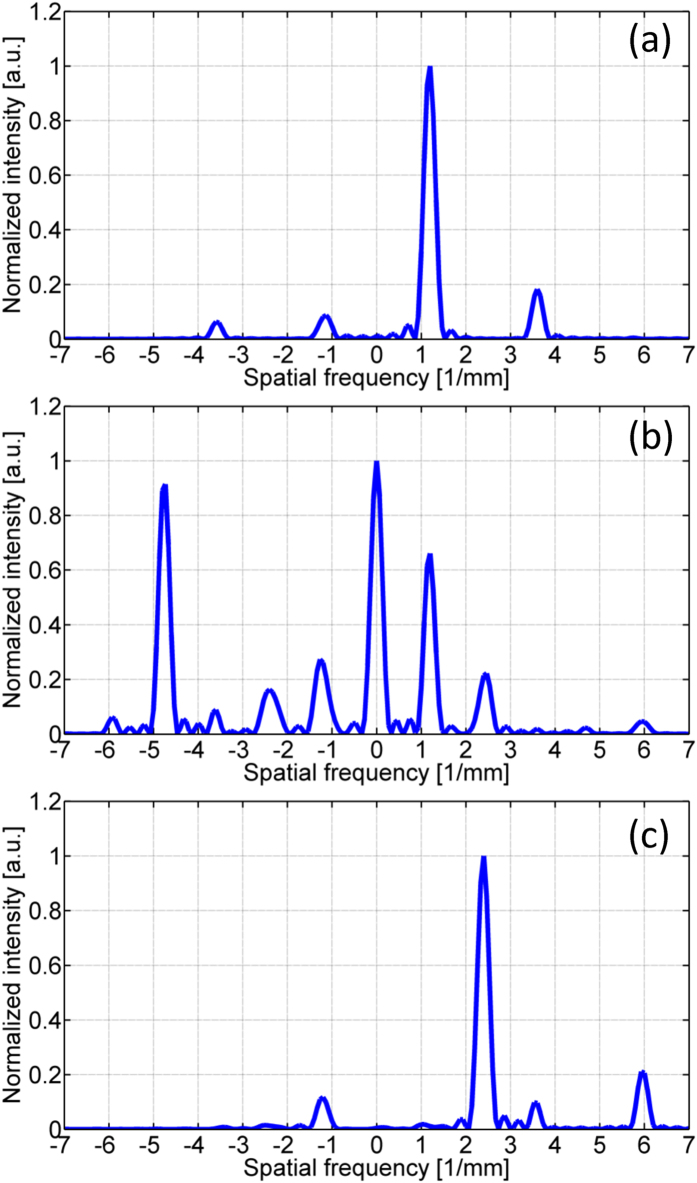
Fourier analysis of the complex field on section 4: (**a)** 1-by-4 splitter, (**b**) 1-by-5 splitter, (**c**) 1-by-6 splitter.

We perform a Fourier analysis of the complex field exp[−(θ_y_ − θ_x_)] generated by section 4 for the case of a 1-by-4 splitter, a 1-by-5 splitter and a 1-by-6 splitter and plot the normalized intensity in Fig. 12(a) to (c) respectively. The Fourier analysis indicate that to generate the transmitted field of the 1-by-5 splitter and the 1-by-6 splitter, phase patterns with higher spatial frequency components are required to be loaded on section 4 of the SLM panels. The complex optical field generated by section 4 of the SLM panels contains frequencies around or above the cutoff frequency of the 4 f systems and cannot be properly relayed. Other factors that may affect the performance of beam splitting with this system include the limited fill factor (87%) of the SLM panels, the phase inaccuracy of the SLM panels, and the lateral and longitudinal misalignment.

It is worthy of noting that the purpose of the experiments is to generate the transmitted complex field of the fan-out polarization grating and demonstrate the validity of the polarization grating design. The physical realization of such polarization gratings should be achieved with the use of metasurfaces^18^,^19^ or form-birefringence structures^20^ that accomplish the spatial polarization modulation not in a cascaded way but in one step. Therefore, high efficiency, great uniformity and accurate polarization state control of individual split beams are expected to approach the theory without suffering from the problem in the 4 f relay systems. The number of split beams is also arbitrary.

In conclusions, we propose and experimentally demonstrate the validity of the scheme of a highly efficient fan-out polarization grating. All the three space-variant parameters of the polarization grating are simultaneously optimized to achieve the function of multi-beam splitting. Besides the high fan-out efficiency, the novel design enables the possibility to control of the polarization states of individual split beams. This polarization grating is promising to find applications in Dammann grating based laser beam combining systems.

## Methods

### SLM panels

The two SLM panels are manufactured by Holoeye Photonics. The model is HEO 1080 P featuring a resolution of 1920 × 1080 with pixel pitch of 8 μm and fill factor of 87%. Since each SLM is divided to two sections, the resolution of each section is 960 × 1080.

The two SLM panels are simultaneously and independently controlled by one computer. The phase patterns of the SLM panels are coded in the red and green color and the built-in function *imwrite* in Matlab is utilized to generate the overall phase pattern to control the system.

The maximum phase retardation provided by the SLM for the wavelength of 632.8 nm is 2.32π. The actual phase patterns implemented on the SLM is the remainder after division of the phase values (shown in Figs 3, 5, 7 and 10) by 2π.

### Simulation method

The optimization process of designing the fan-out polarization grating is accomplished in Matlab with the built-in simulated annealing function: *simulannealbnd*.

## Additional Information

**How to cite this article**: Wan, C. *et al*. Experimental Demonstration of a Highly Efficient Fan-out Polarization Grating. *Sci. Rep.* **6**, 39626; doi: 10.1038/srep39626 (2016).

**Publisher's note:** Springer Nature remains neutral with regard to jurisdictional claims in published maps and institutional affiliations.
